# Novel Halogenated Pyrazine-Based Chalcones as Potential Antimicrobial Drugs †

**DOI:** 10.3390/molecules21111421

**Published:** 2016-10-27

**Authors:** Marta Kucerova-Chlupacova, Veronika Vyskovska-Tyllova, Lenka Richterova-Finkova, Jiri Kunes, Vladimir Buchta, Marcela Vejsova, Pavla Paterova, Lucia Semelkova, Ondrej Jandourek, Veronika Opletalova

**Affiliations:** 1 Department of Pharmaceutical Chemistry and Pharmaceutical Analysis, Faculty of Pharmacy in Hradec Kralove, Charles University in Prague, Heyrovskeho 1203, 500 05 Hradec Kralove, Czech Republic; good-message@seznam.cz (V.V.-T.); lenka.finkova@seznam.cz (L.R.-F.); semelkol@faf.cuni.cz (L.S.); jando6aa@faf.cuni.cz (O.J.); opletalova@faf.cuni.cz (V.O.); 2 Department of Inorganic and Organic Chemistry, Faculty of Pharmacy in Hradec Kralove, Charles University in Prague, Heyrovskeho 1203, 500 05 Hradec Kralove, Czech Republic; kunes@faf.cuni.cz; 3 Department of Biological and Medical Sciences, Faculty of Pharmacy in Hradec Kralove, Charles University in Prague, Heyrovskeho 1203, 500 05 Hradec Kralove, Czech Republic; vejsova@faf.cuni.cz; 4 Department of Clinical Microbiology, University Hospital Hradec Kralove, Sokolska 581, 500 05 Hradec Kralove, Czech Republic; vladimir.buchta@fnhk.cz (V.B.); pavla.paterova@fnhk.cz (P.P.)

**Keywords:** pyrazine, chalcone, halogenated, antifungal, antibacterial, antimycobacterial

## Abstract

Chalcones, i.e., compounds with the chemical pattern of 1,3-diphenylprop-2-en-1-ones, exert a wide range of bio-activities, e.g., antioxidant, anti-inflammatory, anticancer, anti-infective etc. Our research group has been focused on pyrazine analogues of chalcones; several series have been synthesized and tested in vitro on antifungal and antimycobacterial activity. The highest potency was exhibited by derivatives with electron withdrawing groups (EWG) in positions 2 and 4 of the ring B. As halogens also have electron withdrawing properties, novel halogenated derivatives were prepared by Claisen-Schmidt condensation. All compounds were submitted for evaluation of their antifungal and antibacterial activity, including their antimycobacterial effect. In the antifungal assay against eight strains of selected fungi, growth inhibition of *Candida glabrata* and *Trichophyton interdigitale* (formerly *T. mentagrophytes*) was shown by non-alkylated derivatives with 2-bromo or 2-chloro substitution. In the panel of selected bacteria, 2-chloro derivatives showed the highest inhibitory effect on *Staphylococcus* sp. In addition, all products were also screened for their antimycobacterial activity against *Mycobacterium tuberculosis* H37RV My 331/88, *M. kansasii* My 235/80, *M. avium* 152/80 and *M. smegmatis* CCM 4622. Some of the examined compounds, inhibited growth of *M. kansasii* and *M. smegmatis* with minimum inhibitory concentrations (MICs) comparable with those of isoniazid.

## 1. Introduction

**Figure 1:**
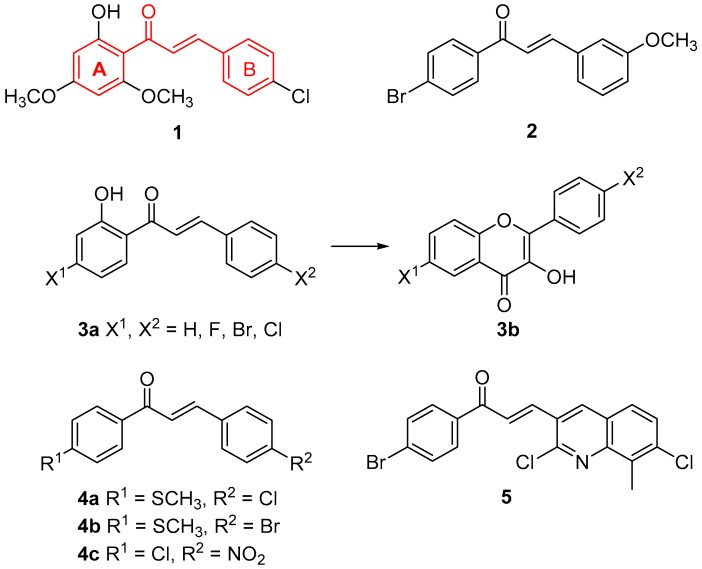
Examples of antifungal and antibacterial chalcone derivatives.

Chalcones are compounds with the basic scaffold of 1,3-diphenylprop-2-en-1-one, containing ring A and ring B connected by an α,β-unsaturated keto linker (indicated in red in Figure 1, structure **1**). Naturally-occurring chalcones (in the plant kingdom) usually bear hydroxy-, methoxy- or prenyl substitutions and might serve as precursors of other flavonoid groups. Chalcones exert a wide range of bio-activities (e.g., antioxidant, anti-inflammatory, anticancer, anti-infective etc.), which were several times reviewed [1,2,3]. In synthetic chalcones, substitutents can be more varied and benzene rings have been many times replaced with other aryls or heteroaryls [2,3].

Based on our previous results concerning chalcones and the positive effect of electron-withdrawing group (EWG) substitution on their antimicrobial effects [4,5], we decided to prepare pyrazine analogues of chalcones halogenated in the ring B. Substitution of chalcones with halogens emerges also in studies of other authors, e.g., compound **1** inhibited growth of *Trichophyton rubrum* at minimum inhibitory concentrations (MIC) 12.5 μg/mL [6]. Compound **2** exerted better antifungal activity against dermatophytes (MIC 0.5–25 μg/mL) than amphotericin B and ketoconazole [7]. In a study comparing antifungal activity of mono- and dihalogenated 1-(2-hydroxyphenyl)-3-phenylprop-2-en-1-ones (**3a**) and their corresponding flavonols (**3b**) [8], it was found that chalcones are more active against *Trichophyton longifusus*, *Aspergillus flavus* and *Microsporum canis* than the flavonols. The most active compound was fluorinated in position 4 of the ring B. Chalcones **4a**–**4c** with halogen substitution in position 4 either in the ring A or in the ring B inhibited growth of *Candida tropicalis* and *Aspergillus flavus* at a concentration of 2 mg/mL at least by 98% [9].

**Figure 2:**
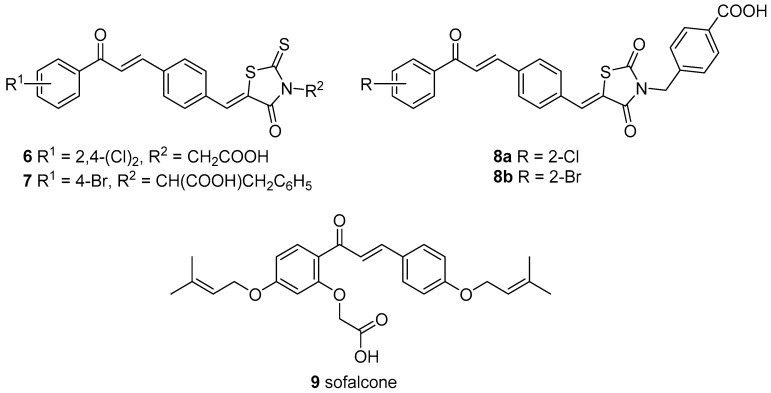
Examples of antibacterial chalcone derivatives.

Antibacterial effects of chalcones have also been reviewed [10,11,12,13] and importance of searching for new antimicrobial agents due to increasing antibiotic resistance of bacteria and fungi has been highlighted [13]. The substitution of aromatic rings with EWG was emphasized in a quinoline-based series of chalcones in association with antibacterial activity. The most active compound, inhibiting growth of *Bacillus subtilis*, *Staphylococcus aureus*, *Streptococcus pyogenes*, *Escherichia coli*, *Klebsiella aerogenes* and *Salmonella typhimurium,* better than chloramphenicol or ciprofloxacin, is depicted in structure **5**. Supposed mechanism of action was explored in a bacterial gyrase assay [14]. Two potentially antibacterial entities were combined in several series containing halogenated chalcones and variously substituted 2-thioxothiazolidin-4-ones (rhodanines) [15,16] or thiazolidin-2,4-dione [17]. Compounds, that inhibited *Staphylococcus* sp. comparably to norfloxacin, are drawn as structure **6** [15] and **7** [16] in Figure 2. In the same research group, analogical chalcones with substituted thiazolidinediones **8a** and **8b** were synthesized (Figure 2). They inhibited growth of two strains of *S. aureus* similarly to oxacillin and norfloxacin [17].

Halogen substitution has been found to be favorable in antimycobacterial diphenylpropenones [18] and 3-phenyl-1-pyridylpropenones [19]. Chalcones including halogenated derivatives have been described as inhibitors of *Mycobacterium tuberculosis* phosphatases [20] or inhibitors of fatty acid synthase II [21].

Halogenated chalcones are further mentioned in literature as potential antimalarial [22], anti-inflammatory [23,24,25], anti-cancer [26] agents, and as potential modulators of multidrug resistance [27].

Sofalcone, explored and marketed in Japan, is a chalcone derivative. Mechanism of its action in ulcer disease is complex, but it possesses bactericidal effect against *Helicobacter pylori* as well [28].

## 2. Results and Discussion

### 2.1. Synthesis

**Scheme 1:**
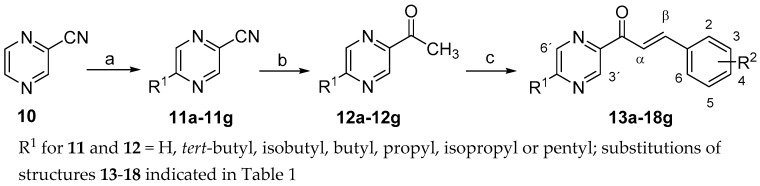
Synthesis of halogenated pyrazine-based chalcones. *Reagents and conditions*: (a) aliphatic acid, AgNO_3_, (NH_4_)_2_S_2_O_8_, water, 80 °C; (b) CH_3_MgI, Et_2_O; (c) halogenated benzaldehyde, pyridine, Et_2_NH.

Preparation of the title compounds is shown in Scheme 1. Pyrazine-2-carbonitrile (**10**) was used as the starting material. Syntheses of intermediates **11a**–**11g** and **12a**–**12g** were accomplished by formerly described methods [29,30]. Acetylpyrazine **12a** and its 5-alkylated congeners **12b**–**12g** served as starting materials for Claisen-Schmidt condensation with 2- or 4-halogenated aromatic aldehydes affording (2*E*)-1-(5-unsubstituted or 5-alkyl-pyrazin-2-yl)-3-(halogenphenyl)prop-2-en-1-ones **13a**–**18g** (substitutions indicated in Table 1), according to a previously reported method [31]. Column chromatography, in several cases repeated, was necessary for the separation of the product from the reaction mixture. To obtain analytically pure crystals, recrystallizations from absolute ethanol were needed, which finally resulted in quite low yields.

Due to difficult purification, only 5 fluorinated derivatives (**13a**, **13b**, **13e**, **14a** and **14b**) were obtained in sufficient amounts. In 2-chloro substituted series, two compounds **15a** and **15b** were successfully purified and available for biological evaluation. Compounds with chlorine in position 4 of the ring B (**16a**–**16f**) were synthesized earlier [5,31]. Influence of compounds **16a**–**16e** on *M. tuberculosis* H37RV (ATCC 27294) and photosynthetic processes has already been published [32]. However, the accomplishment of their antifungal and antibacterial tests failed due to poor solubility in testing media. For the purpose of the present study, these compounds were newly adjusted by rubbing to be able to pass biological assays. Compound **16f** was reported in our previous paper [5]. It displayed neither antifungal nor antimycobacterial activity against *M. tuberculosis* H37RV (ATCC 27294) [5]. In the present study, we report the results of its antibacterial testing and its effects on the growth of other mycobacteria. All 2-brominated compounds **17a**–**17f** were obtained without difficulties in acceptable yields (20%–50%). In contrast, 4-brominated derivatives **18a**–**18g** were mostly obtained in small amounts (yields: 2%–6%). (2*E*)-3-(4-bromophenyl)-1-(5-pentylpyrazin-2-yl)prop-2-en-1-one **18g** is the only representative with a longer alkyl in the pyrazine ring since the longer alkyl the more difficult separation of the product from the reaction mixture. This has been observed for both 2-alkylated pyrazine-2-carbonitriles **12** [29,30] and halogenated (2*E*)-3-phenyl-1-(pyrazin-2-yl)prop-2-en-1-ones reported here.

Identity of compounds was confirmed by melting points, NMR and IR spectra. Proton NMR spectra clearly showed (*J_HαHβ_* = xx − yy Hz) that *E*-isomers have been obtained in all cases. Purity of the compounds was verified by TLC and elemental analysis.

### 2.2. Evaluation of In Vitro Antifungal Activity

**Figure 3:**
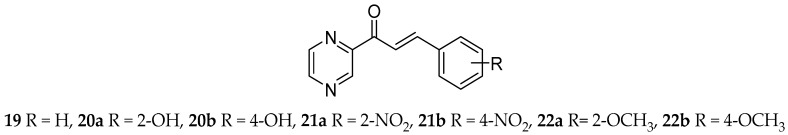
Structure of pyrazine-based chalcones comprised in Table 2 for comparison of antifungal activity.

All prepared compounds, including **16a**–**16e**, were subjected to antifungal assay, only the most active ones are displayed in Table 2. In the panel of selected fungi, inhibition of growth of *Trichophyton interdigitale* 445 by the non-alkylated compounds was the most notable. That is in accordance with the previous results of our research group in the field of antifungal activity of pyrazine analogues of chalcones [4,5,31]. The derivative with 2-chloro substitution in the phenyl, i.e., ring B (**15a**) proved the best activity (MIC 3.9 μmol/L after 24 h incubation), comparable with that of systematically used antimycotic fluconazole (MIC 6.51 μmol/L after 24 h incubation). However, the derivative **15a** did not reach the activity of terbinafine (MIC 0.01–1.72 μmol/L), that is usually used for therapy of dermatomycosis. 4-Fluoro, 2-bromo or 4-bromo substitution (**14a**, **17a** and **18a**) had also inhibiting effect on growth of *T. interdigitale* (MIC 3.9–7.81 μmol/L after 24 h incubation). Halogenated derivatives suppressed the growth of *Candida* spp. as well. In case of 4-fluoro derivative (**14a**), it was a moderate, non-specific inhibition (MIC 31.25–62.5 μmol/L after 24 h incubation), whereas chlorinated derivatives **15a** and **16a** inhibited specifically growth of *Candida glabrata* 20/I and *Candida krusei* E 28 (in both cases MIC 7.81 μmol/L after 24 h incubation). The effect of **15a** on *C. glabrata* (MIC 7.81 μmol/L after 24 h incubation) is better than the effect of clinically used antimycotics listed in Table 2. Comparison with previously prepared substances (**19**–**22**) is provided as well. Upper mentioned active halogenated derivatives were in common more antifungal effective than hydroxy (**20a** and **20b**), nitro (**21a** and **21b**) or methoxy (**22a** and **22b**) derivatives, with the exception of 2-nitro derivative (**21a**) inhibiting strongly *Candida* spp. (antifungal activity in Table 2, structures in Figure 3).

### 2.3. Evaluation of In Vitro Antibacterial Activity

All prepared compounds, including **16a**–**16f** were subjected to antibacterial assay, and only the most active ones are displayed in Table 3. From the panel of tested bacteria, *Staphylococcus* spp. was the most susceptible. Growth of *Staphylococcus aureus* CCM 4516/08 and *S. aureus* H 5996/08 was inhibited by most compounds listed in Table 2 with moderate effect, but growth of *Staphylococcus epidermidis* H6966/08 was inhibited more significantly by 2-chlorinated derivatives (MIC of **15a**: 3.9 μmol/L after 24 h incubation and MIC of **15b:** 7.81 μmol/L after 24 h incubation) than by standard antibiotics. Comparing the character of halogen substitution, fluorine does not seem to be as important in our series as in diphenylpropenones [37]. In another study, focused on diphenylpropenones, derivatives with 3,5-dibromo or 3,5-bis(trifluoromethyl) substitution were the most active compounds [38]. In agreement with the positive effect of chlorine substitution in our series, the most antifungal and antibacterial substance in a 3-quinolinyl-1-thienyl propenones had so far three chlorine atoms [39]. Polyhalogenation of 3-phenyl-1-pyrrol-2-ylpropenones also positively reflected in their antimicrobial activity [40]. In a thiazole-based series of chalcones, derivatives chlorinated in the ring B exerted also better antibacterial and antifungal activity than unsubstituted derivative or nitro derivatives [41]. However, pyrimidine analogues of chalcones, variable halogenated in position 2 of the ring B, exert no remarkable antibacterial or antifungal activity [42].

### 2.4. Evaluation of In Vitro Antimycobacterial Activity

As far as the antimycobacterial screening is concerned, four strains were tested in total (all results are shown Table 4). The most susceptible strain to inhibition by halogenated pyrazine-based analogues of chalcones seems to be *Mycobacterium kansasii* Hauduroy CNCTC My 235/80. However, the lowest MICs have been achieved in 2-chlorinated compounds (**15a** and **15b**) during testing on *Mycobacterium tuberculosis* H37RV CNCTC My 331/88 (MIC 6.25 μg/mL for **15a** and 3.13 μg/mL for **15b**). The efficacy on *M. tuberculosis* in these two cases correlates well with results of in-house testing on *Mycobacterium smegmatis* CCM 4622 (ATCC 607) (MIC 7.81 μg/mL for **15a** and 3.9 μg/mL for **15b**). In comparison with our previous results [4,5,31], positive influence of *tert*-butyl substitution in the position 5 of ring A has been confirmed again during testing on *M. tuberculosis*, which is the most pathogenic strain. As for substitution of ring B, 2-halogen substituent seems to be of importance, as products **13a**, **15b** and **17a** showed the lowest MICs (3.13–6.25 μg/mL against *M. tuberculosis* H37RV CNCTC My 331/88).

In Table 4, calculated lipophilicities of all tested compounds are provided. They do not seem to correlate with biological activity of the compounds. Conformation of particular molecules influenced by halogen position may play a more important role.

## 3. Experimental Section Materials and Methods

### 3.1. Chemistry

#### 3.1.1. Materials and Methods

Pyrazine-2-carbonitrile, 2-fluorobenzaldehyde, 4-fluorobenzaldehyde, 2-chlorobenzaldehyde, 2-bromobenzaldehyde and 4-bromobenzaldehyde were used as starting compounds and were purchased from Sigma-Aldrich (Prague, Czech Republic).

5-Alkylated pyrazine-2-carbonitriles and 5-alkylated pyrazin-2-ylethan-1-ones were prepared from pyrazinecarbonitrile according to previously published procedures [29,30] and identity of these intermediates was confirmed by TLC.

Silica gel 0.040–0.063 nm (Merck, Darmstadt, Germany) and Silpearl (Kavalier, Votice, Czech Republic) were used for flash column chromatography. The purity of the products was checked by TLC on aluminium sheets, silica gel 60 F_254_ (Merck). Mixtures of hexane and ethyl acetate were used for TLC and column chromatography. Analytical samples were dried over anhydrous phosphorus pentoxide under reduced pressure at room temperature. Melting points were determined either on a Boëtius apparatus or on Stuart SMP 20 (Bibby Scientific Ltd., Stone, UK) and are uncorrected. Elemental analyses were performed on an EA 1110 CHNS instrument (CE Instruments, Milano, Italy) or on a Vario Micro Cube Elemental Analyzer (Elementar Analysensysteme GmbH, Hanau, Germany). Infrared spectra were recorded either in KBr pellets on a Nicolet Impact 400 IR spectrophotometer (Thermo Scientific, Waltham, MA, USA) or on germanium crystal using ATR method (indicated at particular compound spectra) on a Nicolet Impact 6700 IR spectrophotometer (Thermo Scientific). Characteristic wavenumbers are given in cm^−1^. ^1^H- and ^13^C-NMR spectra were recorded at ambient temperature on a Varian Mercury-Vx BB 300 spectrometer (Varian Corp., Palo Alto, CA, USA) operating at 300 MHz for ^1^H and 75 MHz for ^13^C or on a VNMR S500 (500 MHz for ^1^H-NMR a 125 MHz for ^13^C-NMR). Chemical shifts were recorded as δ values in ppm, and were indirectly referenced to tetramethylsilane (TMS) via the solvent signal (2.49 for ^1^H, 39.7 for ^13^C in DMSO-d6 and 7.26 for ^1^H, 77.0 for ^13^C in CDCl_3_). Coupling constants *J* are given in Hz.

#### 3.1.2. Synthesis of Halogenated (*E*)-1-(Pyrazin-2-yl)-3-phenylprop-2-en-1-ones

1-(Pyrazin-2-yl)ethan-1-one or 1-(5-alkylpyrazin-2-yl)ethan-1-one (0.01 mol) and the corresponding halogenated benzaldehyde (0.01 mol) were dissolved in pyridine (4.4 mL). Diethylamine (0.01 mol) was added, and the reaction mixture was stirred at 80–120 °C for 1 h. After cooling, the mixture was poured into ice water (200 mL), acidified to pH 3 with a few drops of acetic acid, and then refrigerated for 24 h. The separation of crude products from water depended on their character. Solids were filtered off and crystallized from anhydrous ethanol, while oily mixtures were extracted with diethyl ether and subjected to flash chromatography on silica gel. Hexane–ethyl acetate was used as the eluent in an appropriate ratio (70:30 (*v*/*v*), 80:20 (*v*/*v*) or 90:10 (*v*/*v*)). The fractions containing the desired compounds were combined and crystallized from absolute ethanol to obtain analytically pure crystals. Using this procedure, the following compounds were obtained:

*(2E)-3-(2-Fluorophenyl)-1-(pyrazin-2-yl)prop-2-en-1-one* (**13a**). Light yellow powder; yield 18%, m.p. 110–111 °C (subl.) (114–115 °C [43]); IR (ATR-Ge) 1015, 1056, 1334 (pyrazine) 1597 (C=C), 1670 (C=O); ^1^H-NMR (300 MHz, CDCl_3_) δ 9.36 (d, 1H, *J* = 1.4, H-3′), 8.37 (d, 1H, *J* = 2.5, H-6′), 8.70–8.68 (m, 1H, H-5′), 8.23 (d, 1H, *J* = 16.2, β-CH), 8.10 (d, 1H, *J* = 16.2, α-CH), 7.80–7.70 (m, 1H, H-6), 7.45–7.35 (m, 1H, H-4), 7.24–7.08 (2H, H3, H-5); ^13^C-NMR (75 MHz, CDCl_3_) δ 188.6, 161.9 (d, *J* = 255.1), 148.3, 147.5, 144.9, 143.4, 138.0 (d, *J* = 3.2), 132.4 (d, *J* = 8.6), 129.5 (d, *J* = 2.6), 124.5 (d, *J* = 3.7), 122.9 (d, *J* = 11.4), 123.3 (d, *J* = 6.3), 116.3 (d, *J* = 22.1); elem. anal. calcd. for C_13_H_9_FN_2_O (228.23) 68.42% C; 3.97% H; 12.27% N, found 68.20% C; 4.09% H; 12.55% N.

*(2E)-1-(5-tert-Butylpyrazin-2-yl)-3-(2-fluorophenyl)prop-2-en-1-one* (**13b**). Light yellow powder; yield 13%; m.p. 97–99°C; IR (ATR-Ge) 1014, 1237, 1282 (pyrazine) 1604 (C=C), 1671 (C=O); ^1^H-NMR (500 MHz, CDCl_3_) δ 9.27 (s, 1H, H-3′), 8.73 (s, 1H, H-6′), 8.23 (d, 1H, *J* = 16.1, β-CH), 8.08 (d, 1H, *J* = 16.1, α-CH), 7.74 (d, 1H, *J* = 7.6, H-6), 7.42–7.35 (m, 1H, H-4), 7.19 (t, 1H, *J* = 7.6, H-5), 7.015–7.09 (m, 1H, H-3), 1.44 (s, 9H, CH_3_); ^13^C-NMR (125 MHz, CDCl_3_) δ 188.6, 167.7, 161.9 (d, *J* = 255.3), 145.6, 143.3, 139.9, 137.4 (d, *J* = 2.9), 132.1 (d, *J* = 8.9), 129.4 (d, *J* = 3.0), 124.4 (d, *J* = 3.9), 123.0 (d, *J* = 11.8), 122.7 (d, *J* = 5.8), 116.2 (d, *J* = 21.6), 37.1, 30.9; elem. anal. calcd. for C_17_H_17_FN_2_O (284.33) 71.81% C; 6.03% H; 9.85% N, found 71.72% C; 6.36% H; 10.28% N.

*(2E)-3-(2-Fluorophenyl)-1-(5-propylpyrazin-2-yl)prop-2-en-1-one* (**13e**). Light yellow crystals; yield 20%; m.p. 66–67°C; IR (ATR-Ge) 1018, 1232, 1321 (pyrazine) 1603 (C=C), 1672 (C=O), ^1^H-NMR (500 MHz, CDCl_3_) δ 9.26 (s, 1H, H-3′), 8.52 (s, 1H, H-6′), 8.22 (d, 1H, *J* = 16.1, β-CH), 8.08 (d, 1H, *J* = 16.1, α-CH), 7.77–7.72 (m, 1H, H-6), 7.41–7.35 (m, 1H, H-4), 7.18 (t, 1H, *J* = 7.3, H-5), 7.14–7.09 (m, 1H, H-3), 2.88 (t, 2H, *J* = 7.5, CH_2_), 1.87–1.76 (m, 2H, CH_2_), 0.99 (t, 3H, *J* = 7.5, CH_3_), ^13^C-NMR (125 MHz, CDCl_3_) δ 188.6, 161.9 (d, *J* = 255.1), 161.1, 146.0, 144.1, 142.8, 137.5 (d, *J* = 3.0), 132.2 (d, *J* = 8.8), 129.4 (d, *J* = 2.9), 124.4 (d, *J* = 3.9), 123.0 (d, *J* = 10.8), 122.6 (d, *J* = 5.9), 116.2 (d, *J* = 21.5), 37.7, 22.5, 13.7; elem. anal. calcd. for C_16_H_15_FN_2_O (270.30) 71.10% C; 5.59% H; 10.36% N, found 71.15% C; 5.88% H; 10.89% N.

*(2E)-3-(4-Fluorophenyl)-1-(pyrazin-2-yl)prop-2-en-1-one* (**14a**). Yellow crystals; yield 6%; m.p. 120–124 °C; IR (ATR-Ge) 1016, 1058, 1231 (pyrazine), 1589 (C=C), 1671 (C=O), ^1^H-NMR (300 MHz, CDCl_3_) δ 9.37 (s, 1H, H-3′), 8.77 (s, 1H, H-5′), 8.68 (s, 1H, H-6′), 8.11 (d, 1H, *J* = 15.9, β-CH), 7.93 (d, 1H, *J* = 15.9, α-CH), 7.78–7.65 (m, 2H, H-2, H-6), 7.11 (t, 2H, *J* = 8.2, H-3, H-5), ^13^C-NMR (75 MHz, CDCl_3_) δ 188.4, 164.3 (d, *J* = 252.8), 148.3, 147.5, 144.8, 144.3, 143.3, 131.0 (d, *J* = 3.4), 130.9 (d, *J* = 8.6), 119.7 (d, *J* = 2.3), 116.2 (d, *J* = 22.0); elem. anal. calcd. for C_13_H_9_FN_2_O (228.23) 68.42% C; 3.97% H; 12.27% N, found 67.98% C; 4.12% H; 12.17% N.

*(2E)-1-(5-tert-Butylpyrazin-2-yl)-3-(4-fluorophenyl)prop-2-en-1-one* (**14b**). Light yellow powder; yield 15%; m.p. 136–138 °C; IR (ATR-Ge) 1011, 1139, 1228 (pyrazine), 1595 (C=C), 1669 (C=O); ^1^H-NMR (300 MHz, CDCl_3_) δ 9.28 (d, 1H, *J* = 1.5, H-3′), 8.73 (d, 1H, *J* = 1.5, H-6′), 8.10 (d, 1H, *J* = 16.0, β-CH), 7.90 (d, 1H, *J* = 16.0, α-CH), 7.75–7.66 (m, 2H, H-2, H-6), 7.11 (t, 2H, *J* = 8.7, H-3, H-5), 1.45 (s, 9H, CH_3_); ^13^C-NMR (75 MHz, DMSO) δ 188.5, 167.7, 164.2 (d, *J* = 252.3), 145.6, 143.8, 143.3, 139.8, 131.2 (d, *J* = 3.5), 130.8 (d, *J* = 8.6), 120.1 (d, *J* = 2.3), 116.1 (d, *J* = 21.7), 37.1, 29.7; elem. anal. calcd. for. C_17_H_17_FN_2_O (M 284.33) 71.81% C; 6.03% H; 9.85% N, found 71.54% C; 6.22% H; 10.16% N.

*(2E)-3-(2-Chlorophenyl)-1-(pyrazin-2-yl)prop-2-en-1-one* (**15a**). Light yellow needles; yield 38%; m.p. 132.4–134.2 °C (127–129 °C [43]; IR (ATR-Ge) 1015, 1060, 1330 (pyrazine), 1599 (C=C), 1668 (C=O), ^1^H-NMR (500 MHz, CDCl_3_) δ 9.39 (d, 1H, *J* = 1.5, H-3′), 8.79 (d, 1H, *J* = 2.5, H-5′), 8.71–8.69 (m, 1H, H-6′), 8.40 (d, 1H, *J* = 16.1, β-CH), 8.17 (d, 1H, *J* = 16.1, α-CH), 7.88 (dd, 1H, *J* = 7.8, *J*=2.0, H-6), 7.47–7.44 (m, 1H, H-3), 7.38–7.30 (m, 2H, H-4, H-5), ^13^C-NMR (125 MHz, CDCl_3_) δ 188.3, 148.3, 147.5, 144.9, 143.3, 141.3, 136.0, 133.0, 131.6, 130.3, 128.0, 127.1, 122.4 elem. anal. calcd. for C_13_H_9_ClN_2_O (M 244.68) 63.82% C; 3.71% H; 11.45% N, found 63.76% C; 3.78% H; 11.42% N.

*(2E)-1-(5-tert-Butylpyrazin-2-yl)-3-(2-chlorophenyl)prop-2-en-1-one* (**15b**). Light yellow powder; yield 16%; m.p. 99.8–101.0 °C IR (ATR-Ge) 1014, 1139, 1272 (pyrazine), 1598 (C=C), 1670 (C=O); ^1^H-NMR (300 MHz, CDCl_3_) δ 9.30 (d, 1H, *J* = 1.5, H-3′), 8.74 (d, 1H, *J* = 1.5, H-6′), 8.38 (d, 1H, *J* = 15.9, β-CH), 8.16 (d, 1H, *J* = 15.9, α-CH), 7.87 (d, 1H, *J* = 7.3, *J* = 2.0, H-6), 7.47–7.43 (m, 1H, H-3), 7.37–7.29 (m, 2H, H-4, H-5), 1.49 (s, 9H, CH_3_); ^13^C-NMR (75 MHz, DMSO) δ 188.4, 167.8, 145.6, 143.4, 140.7, 139.8, 135.9, 133.1, 131.4, 130.3, 128.0, 127.0, 122.8, 37.1, 29.7; elem. anal. calcd for C_17_H_17_ClN_2_O (300.79) 67.88% C; 5.70% H; 9.31% N, found 68.18% C; 5.65% H; 9.42% N.

*(2E)-3-(2-Bromophenyl)-1-pyrazin-2-yl)prop-2-en-1-one* (**17a**). Light yellow crystals; yield 50%; m.p. 130.0–133.0 °C (128–130 °C [43]); IR (KBr) 1016, 1029, 1059, 1330 (pyrazine) 1601 (C=C), 1665 (C=O); ^1^H-NMR (300 MHz, CDCl_3_) δ 9.38 (1H, d, *J* = 1.5, H-3′), 8.78 (1H, d, *J* = 2.5, H-5′), 8.69 (1H, dd, *J* = 2.5, *J* = 1.5, H-6′), 8.35 (1H, d, *J* = 16.1, β-H), 8.11 (1H, d, *J* = 16.1, α-H), 7.85 (1H, dd, *J* = 8.0, *J* = 1.6, H-3), 7.64 (1H, dd, *J* = 8.0, *J* = 1.6, H-6), 7.40–7.33 (1H, m, H-4), 7.30–7.22 (1H, m, H-5); ^13^C-NMR (75 MHz, CDCl_3_) δ 188.2, 148.2, 147.6, 144.9, 143.9, 143.3, 134.7, 133.6, 131.7, 128.1, 127.7, 126.5, 122.6; elem. anal. calcd. for C_13_H_9_BrN_2_O (M 289.13) 54.00% C; 3.14% H; 9.69% N, found 53.59% C; 3.27% H; 9.74% N.

*(2E)-3-(2-Bromophenyl)-1-(5-tert-butylpyrazin-2-yl)prop-2-en-1-one* (**17b**). Light yellow crystals; yield 40%; m.p. 91.0–94.5 °C; IR (KBr) 1015, 1040, 1316 (pyrazine), 1597 (C=C), 1669 (C=O); ^1^H-NMR (300 MHz, CDCl_3_) δ 9.29 (1H, d, *J* = 1.5, H-3′), 8.73 (1H, d, *J* = 1.5, H-6′), 8.33 (1H, d, *J* = 16.1, β-H), 8.11 (1H, d, *J* = 16.1, α-H), 7.85 (1H, dd, *J* = 7.7, *J* = 1.5, H-3), 7.64 (1H, dd, *J* = 7.7, *J* = 1.5, H-6), 7.40–7.32 (1H, m, H-4), 7.25 (1H, td, *J* = 7.7, *J* = 1.5, H-5), 1.45 (9H, s, CH_3_); ^13^C-NMR (75 MHz, CDCl_3_) δ 188.3, 167.8, 145.6, 143.4, 143.3, 139.8, 134.9, 133.6, 131.6, 128.1, 127.7, 126.4, 123.0, 37.1, 29.7; elem. anal. calcd. for C_17_H_17_BrN_2_O (345.23) 59.14% C; 4.96% H; 8.11% N, found 59.05% C; 5.13% H; 8.21% N.

*(2E)-3-(2-Bromophenyl)-1-(5-isobutylpyrazin-2-yl)prop-2-en-1-one* (**17c**). Light yellow crystals; yield 29%; m.p. 96.0–100.0 °C; IR (KBr) 1019, 1049, 1309 (pyrazine), 1598 (C=C), 1698 (C=O); ^1^H-NMR (300 MHz, CDCl_3_) δ 9.29 (1H, d, *J* = 1.4, H-3′), 8.49 (1H, d, *J* = 1.4, H-6′), 8.33 (1H, d, *J* = 15.9, β-H), 8.11 (1H, d, *J* = 15.9, α-H), 7.85 (1H, dd, *J* = 7.9, *J* = 1.7, H-3), 7.64 (1H, dd, *J* = 7.9, *J* = 1.7, H-6), 7.40–7.31 (1H, m, H-4), 7.25 (1H, td, *J* = 7.9, *J* = 1.7, H-5), 2.78 (2H, d, *J* = 6.9, CH_2_), 2.27–2.08 (1H, m, CH), 0.97 (6H, d, *J* = 6.9, CH_3_); ^13^C-NMR (75 MHz, CDCl_3_) δ 188.3, 160.6, 145.9, 144.2, 143.4, 143.3, 134.9, 133.6, 131.6, 128.2, 127.6, 126.4, 123.0, 44.8, 29.1, 22.3; elem. anal. calcd. for C_17_H_17_BrN_2_O (M 345.24): 59.14% C; 4.96% H; 8.11% N, found 59.31% C; 5.02% H; 8.42% N.

*(2E)-3-(2-Bromophenyl)-1-(5-butylpyrazin-2-yl)prop-2-en-1-one* (**17d**). Light yellow crystals; yield 20%; m.p. 83.0–88.0 °C; IR (KBr) 1019, 1051, 1320 (pyrazine), 1602 (C=C), 1674 (C=O); ^1^H-NMR (300 MHz, CDCl_3_) δ 9.28 (1H, d, *J* = 1.2, H-3′), 8.52 (1H, d, *J* = 1.2, H-6′), 8.33 (1H, d, *J* = 15.8, β-H), 8.10 (1H, d, *J* = 15.8, α-H), 7.85 (1H, dd, *J* = 7.6, *J* = 1.3, H-3), 7.63 (1H, dd, *J* = 7.6, *J* = 1.3, H-6), 7.39–7.32 (1H, m, H-4), 7.25 (1H, dt, *J* = 7.6, *J* = 1.3, H-5), 2.91 (2H, t, *J* = 7.6, CH_2_), 1.85–1.69 (2H, m, CH_2_), 1.50–1.33 (2H, m, CH_2_), 0.96 (3H, t, *J* = 7.6, CH_3_); ^13^C-NMR (75 MHz, CDCl_3_) δ 188.3, 161.5, 145.9, 144.1, 143.4, 142.8, 134.9, 133.5, 131.6, 128.1, 127.6, 126.4, 123.0, 35.5, 31.4, 22.4, 13.8; elem. anal. calcd. for C_17_H_17_BrN_2_O (M 345.24) 59.14% C; 4.96% H; 8.11% N, found 59.20% C; 5.34% H; 8.29% N.

*(2E)-3-(2-Bromophenyl)-1-(5-propylpyrazin-2-yl)prop-2-en-1-one* (**17e**). Light yellow crystals; yield 24%; m.p. 105.0–107.0 °C; IR (KBr) 1018, 1059, 1317 (pyrazine) 1603 (C=C), 1672 (C=O); ^1^H-NMR (300 MHz, CDCl_3_) δ 9.28 (1H, d, *J* = 1.4, H-3′), 8.52 (1H, d, *J* = 1.4, H-6′), 8.32 (1H, d, *J* = 15.9, β-H), 8.10 (1H, d, *J* = 15.9, α-H), 7.85 (1H, dd, *J* = 7.8, *J* = 1.6, H-3), 7.63 (1H, dd, *J* = 7.8, *J* = 1.6, H-6), 7.39–7.32 (1H, m, H-4′′), 7.25 (1H, td, *J* = 7.8, *J* = 1.6, H-5), 2.89 (2H, t, *J* = 7.4, CH_2_), 1.91–1.74 (2H, m, CH_2_), 1.00 (3H, d, *J* = 7.4, CH_3_); ^13^C-NMR (75 MHz, CDCl_3_) δ 188.3, 161.2, 146.0, 144.1, 143.4, 142.8, 134.9, 133.5, 131.6, 128.1, 127.6, 126.4, 123.0, 37.7, 22.6, 13.8; elem. anal. calcd. for C_16_H_15_BrN_2_O (M 331.21) 58.02% C; 4.57% H; 8.46% N, found 58.01% C; 4.70% H; 8.49% N.

*(2E)-3-(2-Bromophenyl)-1-(5-isopropylpyrazin-2-yl)prop-2-en-1-one* (**17f**). Light yellow needles; yield 33%; m.p. 71.0–73.0 °C; IR (KBr) 1016, 1313 (pyrazine) 1602 (C=C), 1668 (C=O); ^1^H-NMR (300 MHz, CDCl_3_) δ 9.29 (1H, d, *J* = 1.4, H-3′), 8.55 (1H, d, *J* = 1.4, H-6′), 8.33 (1H, d, *J* = 15.9, β-H), 8.11 (1H, d, *J* = 15.9, α-H), 7.85 (1H, dd, *J* = 7.8, *J* = 1.6, H-3), 7.64 (1H, dd, *J* = 7.8, *J* = 1.6, H-6), 7.39–7.32 (1H, m, H-4′′), 7.25 (1H, td, *J* = 7.8, *J* = 1.6, H-5), 3.32–3.12 (1H, m, CH), 1.38 (6H, d, *J* = 6.9, CH_3_); ^13^C-NMR (75 MHz, CDCl_3_) δ 188.3, 165.8, 146.1, 144.0, 143.4, 141.5, 134.9, 133.6, 131.6, 128.1, 127.7, 126.4, 123.0, 34.4, 22.0; elem. anal. calcd. for C_16_H_15_BrN_2_O (M 331.21) 58.02% C; 4.57% H; 8.46% N, found 58.11% C; 4.57% H; 8.81% N.

*(2E)-3-(4-Bromophenyl)-1-(pyrazin-2-yl)prop-2-en-1-one* (**18a**). Light yellow crystals; yield 5%; m.p. 163.0–168.0 °C; IR (KBr) 1017, 1059, 1332 (pyrazine) 1605 (C=C), 1671 (C=O); ^1^H-NMR (300 MHz, CDCl_3_) δ 9.37 (1H, d, *J* = 1.5, H-3′), 8.78 (1H, d, *J* = 2.5, H-6′), 8.69 (1H, dd, *J* = 2.5, *J* = 1.5, H-5′), 8.17 (1H, d, *J* = 16.1, β-H), 7.89 (1H, d, *J* = 16.1, α-H), 7.61–7.52 (4H, m, H-2, H-3, H-5, H-6); ^13^C-NMR (75 MHz, CDCl_3_) δ 188.4, 148.2, 147.6, 144.9, 144.2, 143.3, 133.6, 132.2, 130.2, 125.3, 120.5; elem. anal. calcd for C_13_H_9_BrN_2_O (289.13) 54.00% C; 3.14% H; 9.69% N, found 53.19% C; 3.58% H; 9.21% N.

*(2E)-3-(4-Bromophenyl)-1-(5-tert-butylpyrazin-2-yl)prop-2-en-1-one* (**18b**). Light yellow powder; yield 2%; m.p. 139.0–142.0 °C; IR (KBr) 1020, 1041, 1302 (pyrazine), 1603 (C=C), 1669 (C=O); ^1^H-NMR (300 MHz, CDCl_3_) δ 9.28 (1H, d, *J* = 1.4, H-3′), 8.73 (1H, d, *J* = 1.4, H-6′), 8.17 (1H, d, *J* = 16.1, β-H), 7.87 (1H, d, *J* = 16.1, α-H), 7.62–7.51 (4H, m, H-2, H-3, H-5, H-6), 1.45 (9H, s, CH_3_); ^13^C-NMR (75 MHz, CDCl_3_) δ 188.5, 167.8, 145.6, 143.6, 143.3, 139.9, 133.8, 132.2, 130.2, 125.1, 120.9, 37.1, 29.7; elem. anal. calcd for C_17_H_17_BrN_2_O (345.24) 59.14% C; 4.96% H; 8.11% N, found 59.34% C; 5.14% H; 8.24% N.

*(2E)-3-(4-Bromophenyl)-1-(5-isobutylpyrazin-2-yl)prop-2-en-1-one* (**18c**). Light yellow needles; yield 3%; m.p. 109.0–111.0 °C; IR (KBr) 1020, 1043, 1304 (pyrazine) 1602 (C=C), 1668 (C=O); ^1^H-NMR (300 MHz, CDCl_3_) δ 9.28 (1H, d, *J* = 1.4, H-3′), 8.49 (1H, d, *J* = 1.4, H-6′), 8.17 (1H, d, *J* = 16.1, β-H), 7.87 (1H, d, *J* = 16.1, α-H), 7.61–7.51 (4H, m, H-2, H-3, H-5, H-6), 2.79 (2H, d, *J* = 6.9, CH_2_), 2.27–2.08 (1H, m, CH), 0.97 (6H, d, *J* = 6.9, CH_3_); ^13^C-NMR (75 MHz, CDCl_3_) δ 188.4, 160.6, 146.0, 144.0, 143.8, 143.3, 133.7, 132.2, 130.2, 125.2, 120.8, 44.7, 29.2, 22.3; elem. anal. calcd. for C_17_H_17_BrN_2_O (345.24) 59.14% C; 4.96% H; 8.11% N, found 58.96% C; 5.21% H; 8.21% N.

*(2E)-3-(4-Bromophenyl)-1-(5-butylpyrazin-2-yl)prop-2-en-1-one* (**18d**). Light yellow crystals; yield 2%; m.p. 115.0–116.0 °C; IR (KBr) 1009, 1071, 1330 (pyrazine), 1600 (C=C), 1669 (C=O); ^1^H-NMR (300 MHz, CDCl_3_) δ 9.27 (1H, d, *J* = 1.4, H-3′), 8.52 (1H, d, *J* = 1.4, H-6′), 8.16 (1H, d, *J* = 16.2, β-H), 7.87 (1H, d, *J* = 16.2, α-H), 7.62–7.51 (4H, m, H-2, H-3, H-5, H-6), 2.92 (2H, t, *J* = 7.5, CH_2_), 1.85–1.70 (2H, m, CH_2_), 1.50–1.34 (2H, m, CH_2_), 0.96 (3H, t, *J* = 7.5, CH_3_); ^13^C-NMR (75 MHz, CDCl_3_) δ 188.4, 161.5, 145.9, 144.1, 143.7, 142.8, 133.8, 132.2, 130.2, 125.1, 120.9, 35.5, 31.4, 22.4, 13.82; elem. anal. calcd. for C_17_H_17_BrN_2_O (345.24) 59.14% C; 4.96% H; 8.11% N, found 59.05% C; 5.33% H; 8.40% N.

*(2E)-3-(4-Bromophenyl)-1-(5-propylpyrazin-2-yl)prop-2-en-1-one* (**18e**). Light yellow crystals; yield 4%; m.p. 99.0–100.0 °C; IR (KBr) 1009, 1045, 1071, 1321 (pyrazine) 1600 (C=C), 1670 (C=O); ^1^H-NMR (300 MHz, CDCl_3_) δ 9.27 (1H, d, *J* = 1.4, H-3′), 8.52 (1H, d, *J* = 1.4, H-6′), 8.16 (1H, d, *J* = 15.9, β-H), 7.87 (1H, d, *J* = 15.9, α-H), 7.61–7.51 (4H, m, H-2, H-3, H-5, H-6), 2.90 (2H, d, *J* = 7.6, CH_2_), 1.91–1.75 (1H, m, CH_2_), 1.01 (3H, t, *J* = 7.6, CH_3_); ^13^C-NMR (75 MHz, CDCl_3_) δ 188.4, 161.2, 146.0, 144.0, 143.7, 142.9, 133.8, 132.2, 130.2, 125.1, 120.8, 37.7, 22.6, 13.8; elem. anal. calcd. for C_16_H_15_BrN_2_O (331.21) 58.02% C; 4.57% H; 8.46% N, found 57.63% C; 4.80% H; 8.57% N.

*(2E)-3-(4-Bromophenyl)-1-(5-isopropylpyrazin-2-yl)prop-2-en-1-one* (**18f**). Light yellow crystals; yield 4%; m.p. 106.0–108.0 °C; IR (KBr) 1017, 1040, 1068, 1334 (pyrazine) 1600 (C=C), 1667 (C=O); ^1^H-NMR (300 MHz, CDCl_3_) δ 9.27 (1H, d, *J* = 1.5, H-3′), 8.56 (1H, d, *J* = 1.5, H-6′), 8.16 (1H, d, *J* = 15.8, β-H), 7.87 (1H, d, *J* = 15.8, α-H), 7.61–7.52 (4H, m, H-2, H-3, H-5, H-6), 3.31–3.15 (1H, m, CH), 1.38 (6H, d, *J* = 7.0, CH_3_);^13^C-NMR (75 MHz, CDCl_3_) δ 188.4, 165.8, 146.2, 143.9, 143.7, 141.5, 133.8, 132.2, 130.2, 125.1, 120.9, 34.3, 22.0; elem. anal. calcd. for C_16_H_15_BrN_2_O (331.21) 58.02% C; 4.57% H; 8.46% N, found 57.92% C; 4.75% H; 8.52% N.

*(2E)-3-(4-Bromophenyl)-1-(5-pentylpyrazin-2-yl)prop-2-en-1-one* (**18g**). Light yellow needles; yield 6%; m.p. 98.0–100.0 °C; IR (KBr) 1010, 1036, 1075, 1320 (pyrazine) 1603 (C=C), 1669 (C=O); ^1^H-NMR (300 MHz, CDCl_3_) δ 9.27 (1H, d, *J* = 1.1, H-3′), 8.52 (1H, d, *J* = 1.1, H-6′), 8.16 (1H, d, *J* = 16.2, β-H), 7.87 (1H, d, *J* = 16.2 Hz, α-H), 7.62–7.51 (4H, m, H-2, H-3, H-5, H-6), 2.91 (2H, t, *J* = 7.4, CH_2_), 1.87–1.71 (2H, m, CH_2_), 1.46–1.28 (4H, m, CH_2_), 0.90 (3H, t, *J* = 7.4, CH_3_); ^13^C-NMR (75 MHz, CDCl_3_) δ 188.4, 161.5, 145.9, 144.1, 143.7, 142.8, 133.8, 132.2, 130.2, 125.1, 120.9, 35.8, 31.4, 29.0, 22.4, 13.9; elem. anal. calcd. for C_18_H_19_BrN_2_O (359.27) 60.18% C; 5.33% H; 7.80% N, found 59.72% C; 5.58% H; 7.88% N.

### 3.2. Biological Evaluation

#### 3.2.1. Evaluation of In Vitro Antifungal Activity

The antifungal activity of all compounds was evaluated by the modified microdilution broth CSLI standards [44,45]. The organisms examined included *Candida albicans* ATCC 44859 (American Type Culture Collection, Manassas, VA, USA), *Candida tropicalis* 156, *Candida krusei* E 28, *Candida glabrata* 20/I, *Trichosporon asahii* 1188, *Aspergillus fumigatus* 231, *Lichtheimia corymbifera* 272, and *Trichophyton interdigitale* 445. All strains, except ATCC one tested, are clinical isolates obtained from the Department of Clinical Microbiology, University Hospital and Faculty of Medicine, Charles University, Prague, Czech Republic. Before testing, each strain was subcultured on Sabouraud dextrose agar (SDA; Difco/Becton Dickinson, Detroit, MI, USA) and maintained on the same medium at 4 °C. Fungal inocula were prepared by suspending yeasts, conidia, or sporangiospores in sterile 0.85% saline. The cell density was adjusted using a Bürker’s chamber to yield a stock suspension of 1.0 ± 0.2 × 10^5^ colony forming units (CFU)/mL and 1.0 ± 0.2 × 10^6^ CFU/mL for yeasts and molds, respectively. The final inoculum was made by 1:20 dilution of the stock suspension with the test medium. The compounds were dissolved in DMSO, and the antifungal activity was determined in RPMI 1640 media (KlinLab, Prague, Czech Republic) buffered to pH 7.0 with 0.165 M 3-morpholinopropane-1-sulfonic acid (Sigma-Aldrich, St. Louis, MO, USA). Controls consisted of medium and DMSO alone. The final concentration of DMSO in the test medium did not exceed 1% (*v*/*v*) of the total solution. The concentrations of the studied substances ranged from 500 to 0.488 μmol/L. The minimum inhibitory concentration (MIC), was defined as 80% or greater (for yeasts and yeast-like organisms—IC_80_), resp. 50% or greater (for molds—IC_50_) reduction of growth in comparison with the control. The values of MICs were determined after 24 and 48 h of static incubation at 35 °C. In the case of *T. interdigitale*, the MICs were recorded after 72 and 120 h due to its slow growth rate. Fluconazole and voriconazole were used as reference antifungal drugs.

#### 3.2.2. Evaluation of In Vitro Antibacterial Activity

The antibacterial activity of all compounds was evaluated by the microdilution broth method [46]. The organisms examined included strains from Czech Collection of Microorganisms (Brno, Czech Republic): *Staphylococcus aureus* CCM 4516/08 (SA), *Escherichia coli* CCM 4517 (EC), *Pseudomonas aeruginosa* CCM 1961 (PA). These strains are recommended as standards for testing of antibacterial activities. Other strains were clinical isolates (Department of Clinical Microbiology, University Hospital and Faculty of Medicine in Hradec Králové, Charles University in Prague, Czech Republic): *Staphylococcus aureus* H 5996/08 (methicillin resistant, MRSA), *Staphylococcus epidermidis* H 6966/08 (SE), *Enterococcus* sp. J 14365/08 (EF), *Klebsiella pneumoniae* D11750/08 (KP), *Klebsiella pneumoniae* J 14368/08 (ESBL positive, KP-E). All strains were subcultured on Mueller-Hinton agar (MHA) (Difco/Becton Dickinson, Detroit, MI) at 35 °C and maintained on the same medium at 4 °C. Prior to testing, each strain was passaged onto MHA. Bacterial inocula were prepared by suspending in sterile 0.85% saline. The cell density of the inoculum was adjusted using densitometer to yield suspension of density equivalent 0.5 McFarland scale which is equal to a number of 1. 5 × 10^8^ CFU/mL.

The compounds were dissolved in DMSO, and the antibacterial activity was determined in Mueller-Hinton liquid broth (Difco/Becton Dickinson, Detroit, MI, USA), buffered to pH 7.0. Controls consisted of medium and DMSO alone. The final concentration of DMSO in the test medium did not exceed 1% (*v*/*v*) of the total solution composition. The minimum inhibitory concentration (MIC), defined as 95% inhibition of bacterial growth as compared to control, was determined after 24 and 48 h of static incubation at 35 °C. Neomycin, bacitracin and penicillin have been used as reference antibacterial drugs.

#### 3.2.3. Evaluation of In Vitro Antimycobacterial Activity on *Mycobacterium tuberculosis* H37RV CNCTC My 331/88, *M. kansasii* Hauduroy CNCTC My 235/80 and *M. avium* ssp. *avium* Chester CNCTC My 80/72

Microdilution panel method was used. Tested strains *Mycobacterium tuberculosis* H37RV CNCTC My 331/88, *M. kansasii* Hauduroy CNCTC My 235/80 and *M. avium* ssp. *avium* Chester CNCTC My 80/72 were obtained from Czech National Collection of Type Cultures (CNCTC), National Institute of Public Health, Prague, Czech Republic. Middlebrook 7H9 broth (Sigma-Aldrich, Steinheim, Germany) enriched with 0.4% of glycerol (Sigma-Aldrich) and 10% of OADC supplement (oleic acid, albumin, dextrose, catalase; Himedia, Mumbai, India) of declared pH 6.6 was used for cultivation. Tested compounds were dissolved and diluted in DMSO and mixed with broth (25 μL of DMSO solution in 4.475 mL of broth) and placed (100 μL) into microtitration plate wells. Mycobacterial inocula were suspended in isotonic saline solution and the density was adjusted to 0.5–1.0 McFarland. These suspensions were diluted by 10^−1^ and used to inoculate the testing wells, adding 100 μL of suspension to 100 μL of the DMSO/broth solution of tested compound. Final concentrations of tested compounds in wells were 100, 50, 25, 12.5, 6.25, 3.13 and 1.56 μg/mL. Isoniazid was used as positive control (inhibition of growth). Negative control consisted of broth plus DMSO. 30 μL of Alamar Blue working solution (1:1 mixture of 0.01% resazurin sodium salt (aq. sol.) and 10% aqueous solution of Tween 80) was added usually after 5 days of incubation. Results were then determined after 24 h of incubation and interpreted according to Franzblau [47]. The MIC (μg/mL) was determined as the lowest concentration which prevented the blue to pink colour change.

#### 3.2.4. Evaluation of In Vitro Antimycobacterial Activity on *Mycobacterium smegmatis*

An antimycobacterial assay was performed with fast growing *Mycobacterium smegmatis* CCM 4622 (ATCC 607) from Czech Collection of Microorganisms (Brno, Czech Republic). The technique used for activity determination was microdilution broth panel method using 96-well microtitration plates. Culturing medium was Middlebrook 7H9 (MB) broth (Sigma-Aldrich) enriched with 0.4% of glycerol (Sigma-Aldrich) and 10% of Middlebrook OADC growth supplement (Himedia, Mumbai, India). Tested compounds were dissolved in DMSO (Sigma-Aldrich) then MB broth was added to obtain concentration 2000 µg/mL. Standards used for activity determination were isoniazid (INH), rifampicin (RIF) and ciprofloxacin (CPX) (Sigma-Aldrich). Final concentrations were reached by twofold dilution and addition of mycobacterial suspension and were set as 500, 250, 125, 62.5, 31.25, 15.625, 7.81 and 3.91 µg/mL except to standards ciprofloxacin and rifampicin where the final concentrations were 12.5, 6.25, 3.125, 1.56, 0.78, 0.39, 0.195 and 0.098 µg/mL. Drug-free controls consisted of broth and DMSO and were used as a growth control. The final concentration of DMSO did not exceeded 2.5% (*v*/*v*) to not affect the growth of *M. smegmatis*. Plates were sealed with polyester adhesive film and incubated in the dark at 37 °C without agitation. The addition of 0.01% solution of resazurin sodium salt followed after 48 h. This stain was prepared by dissolving resazurin sodium salt (Sigma-Aldrich) in deionised water to get 0.02% solution. Then 10% aqueous solution of Tween 80 (Sigma-Aldrich) was prepared. Both liquids were mixed up making use of the same volumes and filtered through syringe membrane filter. Microtitration plates were then incubated for further 4 h. Antimycobacterial activity was expressed as MIC and the value was determined on the basis of stain color change (blue color—active compound; pink color—non active compound). MIC values for standards were within the ranges 7.81–15.625 µg/mL for INH, 0.78–1.56 µg/mL for RIF and 0.098–0.195 µg/mL for CPX. All experiments were conducted in duplicate.

### 3.3. Calculation of Lipophilicity

Theoretical lipophilicities logP and corrigated values ClogP of all compounds have been calculated in ChemDraw Professional 15.0, part of ChemOffice (Perkin Elmer, Waltham, MA, USA).

## 4. Conclusions

Chalcones have a very simple chemistry, which enables multiplicity of substitutions with easy synthesis and different pharmacological potential in dependence on particular structural modification [13]. Within this work, twenty halogenated pyrazine-based compounds have been prepared and characterized. They are all novel compounds with exception of three compounds. They were tested on antifungal, antibacterial, as well as on antimycobacterial effects. 4-Chlorinated series, prepared earlier, have been included in the assays. The results of biological screenings have been compared with our previously published compounds. Importance of an EWG substitution has been confirmed and some compounds displayed comparable or even better inhibitory activity than standard antifungals, antibiotics and antimycobacterial agents.

As far as the structure-activity relationships are concerned, halogen substitution in the ring B of pyrazine-based chalcones proved to have positive influence on antifungal effect against *Candida* spp. and *Trichophyton interdigitale* in comparison with our previously prepared series of pyrazine-based chalcones [4,5,31]. Generally, chlorinated and brominated series inhibited growth of fungi more significantly than fluorinated derivatives.

Halogen substitution in the ring B had positive impact on inhibition of *Staphylococcus* spp., whereas it did not influence inhibition of other bacteria from the panel. As for specific substitution, 2-chlorinated derivative inhibited markedly growth of *S. epidermidis*. Concerning mycobacteria, halogenated series inhibited significantly growth of *M. krusei*, *M. tuberculosis* and *M. smegmatis*, but they did not strongly influence the growth of *M. avium*. The inhibiting activity of halogenated series was comparable with that of nitro series prepared previously [4,5]. Derivatives substituted by halogen in position 2 of the ring B and concurrently substituted by *tert*-butyl in the position 5 of the pyrazine ring showed the highest inhibition of *M. tuberculosis*. Importance of *tert*-butyl substitution has been observed previously in other series [5]. Presence of isopropyl in position 5 of the pyrazine ring seems to be important in chlorinated or brominated series in the test against *M. krusei*.

## Figures and Tables

**Table 1:** Halogenated pyrazine-based chalcones **13a**–**18g** tested within this work.

R^2^	R^1^						
	H	*tert*-butyl	isobutyl	butyl	propyl	isopropyl	pentyl
2-F	**13a**	**13b**	-	-	**13e**	-	-
4-F	**14a**	**14b**	-	-	-	-	-
2-Cl	**15a**	**15b**	-	-	-	-	-
4-Cl	**16a** ^a^	**16b** ^a^	**16c** ^a^	**16d** ^a^	**16e** ^a^	**16f** ^b^	-
2-Br	**17a**	**17b**	**17c**	**17d**	**17e**	**17f**	-
4-Br	**18a**	**18b**	**18c**	**18d**	**18e**	**18f**	**18g**

^a^ Synthesis, antimycobacterial activity against *M. tuberculosis* H37RV (ATCC 27294) and influence on photosynthetic processes published previously [32]; ^b^ synthesis, antimycobacterial activity against *M. tuberculosis* H37RV (ATCC 27294) and antifungal activity published previously [5].

**Table 2:** Comparison of antifungal activity of selected halogenated 3-phenyl-1-pyrazin-2-ylprop-2-en-1-ones with ring B-variously substituted analogues and standard antimycotics.

Compd.	Substituent in the Ring B	MIC (μmol/L) *							
		CA	CT	CK	CG	TA	AF	LC	TI
		24 h	24 h	24 h	24 h	24 h	24 h	24 h	72 h
		48 h	48 h	48 h	48 h	48 h	48 h	48 h	120 h
**13a**	2-F	15.63	250	15.62	62.5	125	250	˃500	250
		62.5	˃500	125	125	125	˃500	˃500	250
**14a**	4-F	31.25	62.5	31.25	62.5	62.5	250	˃500	7.81
		62.5	125	62.5	125	250	˃500	˃500	15.62
**15a**	2-Cl	˃125	˃125	31.25	7.81	62.5	˃125	˃125	3.9
		˃125	˃125	62.5	15.62	62.5	˃125	˃125	3.9
**16a**	4-Cl	125	125	7.81	31.25	31.25	62.5	˃125	˃125
		˃125	˃125	15.62	62.5	125	˃125	˃125	˃125
**17a**	2-Br	62.5	˃125	62.5	7.81	62.5	˃125	˃125	7.81
		˃125	˃125	˃125	31.25	125	˃125	˃125	7.81
**18a**	4-Br	15.62	31.25	32.6	˃125	˃125	125	˃125	3.9
		31.25	125	˃125	˃125	˃125	˃125	˃125	7.81
**19 ^a^**	H	15.63	31.25	31.25	31.25	250	125	250	7.81
		31.25	62.5	62.5	62.5	250	125	500	15.63
**20a ^b^**	2-OH	62.5	125	125	125	125	125	125	15.62
		62.5	250	250	250	250	250	250	31.25
**20b**	4-OH	˃250 ^c^	˃250 ^c^	˃250 ^c^	˃250 ^c^	˃250 ^c^	˃250 ^c^	˃250 ^c^	62.5 ^b^
		˃250 ^c^	˃250 ^c^	˃250 ^c^	˃250 ^c^	˃250 ^c^	˃250 ^c^	˃250 ^c^	125 ^b^
**21a**	2-NO_2_	7.81 ^d^	7.81 ^d^	7.81 ^d^	7.81 ^d^	˃125 ^d^	31.25 ^d^	˃125 ^d^	31.25 ^e^
		15.63 ^d^	7.81 ^d^	15.63 ^d^	7.81 ^d^	˃125 ^d^	˃125 ^d^	˃125 ^d^	31.25 ^e^
**21b**	4-NO_2_	31.25 ^f^	˃62.5 ^f^	31.25 ^f^	˃62.5 ^f^	˃62.5 ^f^	˃62.5 ^f^	˃62.5 ^f^	15.63 ^e^
		62.5 ^f^	˃62.5 ^f^	31.25 ^f^	˃62.5 ^f^	˃62.5 ^f^	˃62.5 ^f^	˃62.5 ^f^	15.63 ^e^
**22a ^d^**	2-OCH_3_	31.25	31.25	˃125	31.25	˃125	˃125	˃125	31.25
		125	62.5	˃125	125	˃125	˃125	˃125	125
**22b ^d^**	2-OCH_3_	62.5	125	125	125	500	62.5	500	31.25
		125	250	250	125	˃500	125	˃500	62.5
**Fluconazole**		0.24	˃500	125	41.64	250	˃500	˃500	6.51
		0.24	˃500	250	250	500	˃500	˃500	104
**Voriconazole**		0.005	125	0.65	83.58	3.26	0.49	208	0.08
		0.007	250	1.95	250	14.32	1.3	250	0.12
**Terbinafine**		6.86 ^g^	6.86 ^g^	6.86 ^g^	6.86 ^g^	-	-	-	0.01–0.86 ^g^

* MIC defined as IC_80_ for yeasts and yeast-like organisms and IC_50_ for molds; Notes: **CA** = *Candida albicans* ATCC 44859; **CT** = *Candida tropicalis* 156; **CK** = *Candida krusei* E 28; **CG** = *Candida glabrata* 20/I; **TA** = *Trichosporon asahii* 1188; **AF** = *Aspergillus fumigatus* 231; **LC** = *Lichtheimia corymbifera* 272; **TI** = *Trichophyton interdigitale* 445; ^a^ ref. [32]; ^b^ ref. [31]; ^c^ ref. [33]; ^d^ ref. [34]; ^e^ ref. [4]; ^f^ ref. [35]; ^g^ IC_50_ after 48 h of incubation for yeasts and 7 days of incubation for *T. interdigitale* [36].

**Table 3:** Comparison of antibacterial activity of selected novel halogenated 3-phenyl-1-pyrazin-2-ylprop-2-en-1-ones with standard antibacterial agents.

Compd.	Substituent in the Ring		IC_95_ (μmol/L)							
			SA	MRSA	SE	EF	EC	KP	KP-E	PA
	A	B	24 h	24 h	24 h	24 h	24 h	24 h	24 h	24 h
			48 h	48 h	48 h	48 h	48 h	48 h	48 h	48 h
**13a**	H	2-F	125	250	31.25	500	˃500	˃500	˃500	˃500
			250	250	125	500	˃500	˃500	˃500	˃500
**13e**	5-pro	2-F	125	˃500	31.25	˃500	˃500	˃500	˃500	˃500
			˃500	˃500	125	˃500	˃500	˃500	˃500	˃500
**14a**	H	4-F	125	125	31.25	˃500	˃500	˃500	˃500	˃500
			125	500	62.5	˃500	˃500	˃500	˃500	˃500
**15a**	H	2-Cl	31.25	62.5	3.9	62.5	˃500	˃500	˃500	˃500
			62.5	250	15.62	250	˃500	˃500	˃500	˃500
**15b**	5-*t*-bu	2-Cl	125	˃500	7.81	125	˃500	˃500	˃500	˃500
			500	˃500	31.25	500	˃500	˃500	˃500	˃500
**17a**	H	2-Br	31.25	62.5	62.5	˃500	˃500	˃500	˃500	˃500
			31.25	250	250	˃500	˃500	˃500	˃500	˃500
**Neomycin**			2.60	1.95	9.11	291.67	2.28	1.30	2.28	7.81
			3.25	4.23	13.02	291.67	2.28	1.30	2.28	15.62
**Bacitracin**			10.41	13.02	15.62	31.25	-	-	-	-
			18.23	26.04	31.25	52.08				
**Penicillin**			0.57	83.33	135.42	7.81	125.00	333.33	-	-
			0.73	104.17	208.33	15.62	125.00	416.67		

**SA** = *Staphylococcus aureus* CCM 4516/08; **MRSA** = *Staphylococcus aureus* H 5996/08 methicillin-resistant; **SE** = *Staphylococcus epidermidis* H6966/08; **EF** = *Enterococcu*s sp. J 14365/08; **EC** = *Escherichia coli* CCM4517; **KP** = *Klebsiella pneumoniae* D 11750/08; **KP-E** = *Klebsiella pneumoniae* J 14368/08 (ESBL positive, KP-E); **PA** = *Pseudomonas aeruginosa* CCM 1961.

**Table 4:** Comparison of antimycobacterial activity of halogenated 3-phenyl-1-pyrazin-2-ylprop-2-en-1-ones with standard antimycobacterial agents and calculated lipophilicities of the compounds.

Compd.	Substituent in the Ring		MIC (μg/mL)				Lipophilicity ^a^	
	A	B	MK	MA	MT	MS	LogP	ClogP
**13a**	H	2-F	12.5	100	12.5	15.625	1.49	2.11066
**13b**	5-*t*-bu	2-F	12.5	˃100	6.25	≥500	3.62	3.93666
**13e**	5-pro	2-F	12.5	˃100	12.5	15.625	3.1	3.66767
**14a**	H	4-F	12.5	100	25	15.625	1.49	2.11066
**14b**	5-*t*-bu	4-F	˃25	˃25	˃25	≥125	3.62	3.93666
**15a**	H	2-Cl	25	˃100	6.25	7.81	1.89	2.68067
**15b**	5-*t*-bu	2-Cl	25	˃100	3.13	3.9	4.02	4.50666
**16a**	H	4-Cl	6.25	100	50	15.625	1.89	2.68067
**16b**	5-*t*-bu	4-Cl	˃50	˃50	˃50	≥250	4.02	4.50666
**16c**	5-isobu	4-Cl	12.5	˃25	˃25	≥125	3.83	4.63666
**16d**	5-bu	4-Cl	12.5	12.5	˃50	≥250	3.92	4.76666
**16e**	5-pro	4-Cl	12.5	12.5	25	≥500	3.50	4.23767
**16f**	5-isopro	4-Cl	6.25	˃50	12.5	≥125	3.48	4.10767
**17a**	H	2-Br	12.5	100	25	15.625	2.16	2.83067
**17b**	5-*t*-bu	2-Br	12.5	100	6.25	62.5	4.39	4.65667
**17c**	5-isobu	2-Br	100	˃100	˃100	62.5	4.10	4.78667
**17d**	5-bu	2-Br	25	˃100	25	15.625	4.19	4.91667
**17e**	5-pro	2-Br	6.25	˃100	12.5	≥500	3.77	4.38767
**17f**	5-isopro	2-Br	12.5	50	12.5	15.625	3.75	4.25767
**18a**	H	4-Br	12.5	˃100	12.5	62.5	2.16	2.83067
**18b**	5-*t*-bu	4-Br	12.5	˃50	˃50	≥250	4.29	4.65667
**18c**	5-isobu	4-Br	25	˃100	˃100	250	4.10	4.78667
**18d**	5-bu	4-Br	˃50	˃50	˃50	125	4.19	4.91667
**18e**	5-pro	4-Br	12.5	˃50	50	≥250	3.77	4.38767
**18f**	5-isopro	4-Br	6.25	˃100	25	≥500	3.75	4.25767
**18g**	5-pent	4-Br	˃50	˃50	˃50	≥250	4.61	5.44566
**Isoniazid**			3.13–12.5	3.13–6.25	0.1–0.39	7.81–15.625		
**Rifampicin**			-	-	-	0.78–1.56		
**Ciprofloxacin**			-	-	-	0.098–0.195		

**MK** = *Mycobacterium kansasii* Hauduroy CNCTC My 235/80; **MA** = *M. avium* ssp. *avium* Chester CNCTC My 80/72; **MT** = *M. tuberculosis* H37RV CNCTC My 331/88; **MS** = *Mycobacterium smegmatis* CCM 4622 (ATCC 607); ^a^ lipophilicity calculated by ChemDraw Professional 15.0, part of ChemOffice (Perkin Elmer, Waltham, MA, USA).
